# The Autophagic Flux Inhibitor Bafilomycine A1 Affects the Expression of Intermediary Metabolism-Related Genes in Trout Hepatocytes

**DOI:** 10.3389/fphys.2019.00263

**Published:** 2019-03-18

**Authors:** Sarah Séité, Tracy Pioche, Nicolas Ory, Elisabeth Plagnes-Juan, Stéphane Panserat, Iban Seiliez

**Affiliations:** ^1^INRA, E2S UPPA, UMR 1419, Nutrition, Métabolisme, Aquaculture, University of Pau and Pays de l’Adour, Saint-Pée-sur-Nivelle, France; ^2^Evonik Rexim, Ham, France; ^3^Evonik Nutrition and Care GmbH, Hanau, Germany

**Keywords:** fish, hepatocyte, autophagy, intermediary metabolism, ER stress, gene expression

## Abstract

Autophagy is an evolutionarily conserved process of cellular self-eating which emerged these last years as a major adaptive metabolic response to various stresses such as fasting, hypoxia, or environmental pollutants. However, surprisingly very few data is currently available on its role in fish species which are directly exposed to frequent environmental perturbations. Here, we report that the treatment of fasted trout hepatocytes with the autophagy inhibitor Bafilomycine A1 lowered the mRNA levels of many of the gluconeogenesis-related genes and increased those of genes involved in intracellular lipid stores. Concurrently, intracellular free amino acid levels dropped and the expression of the main genes involved in the endoplasmic reticulum (ER) stress exhibited a sharp increase in autophagy inhibited cells. Together these results highlight the strong complexity of the crosstalk between ER, autophagy and metabolism and support the importance of considering this function in future studies on metabolic adaptation of fish to environmental stresses.

## Introduction

Macroautophagy (autophagy hereafter) is a cellular function conserved in eukaryotes that allows the recruitment of substrates into lysosomes for their degradation (Bento et al., 2016). In addition to its role as a “cell cleaner,” autophagy allows providing energy during fasting or other cellular stress in order to promote survival (Yang and Klionsky, 2010). In mammals, several studies demonstrated that autophagy maintains cellular, and energy homeostasis by degrading and recycling the main energy sources (proteins, lipids or glycogen) on exposure to various stresses (Madrigal-Matute and Cuervo, 2016). One of the first metabolic functions attributed to autophagy has been the release of amino acids through protein degradation during starvation. The released amino acids not only sustain protein synthesis under fasting condition, but also feed the tricarboxylic acid cycle for ATP production (Lum et al., 2005; Rabinowitz and White, 2010; Ezaki et al., 2011; Thomas et al., 2018). Furthermore, autophagic proteolysis in liver has been shown to makes a significant contribution to the maintenance of glycaemia during fasting by releasing amino acids for glucose production via gluconeogenesis (Ezaki et al., 2011). In addition to its role in protein breakdown, autophagy has also been shown to play an important role in the degradation of hepatic lipid stores through a selective form of autophagy termed lipophagy (Singh et al., 2009). During this process, autophagy-dependent breakdown of lipid droplets supplies free fatty acids, which undergo β-oxidation in the mitochondria to support ATP production (Singh et al., 2009; Rambold et al., 2015; Welte, 2015). Although less studied than the two former autophagic processes, lysosomal breakdown of hepatic glycogen might also contribute to glucose homeostasis during some critical periods. In mice, this process known as glycophagy has thus been shown to be necessary to sustain life during the period of postnatal hypoglycemia (Kotoulas et al., 2006). Collectively, these data highlight the critical importance of autophagy for the adaptation of intermediary metabolism to environmental changes.

In fish, this cellular function is attracting growing interest and the number of studies in this field is constantly increasing. As such, induction of autophagy has been demonstrated upon different biotic or abiotic stress situations including pollution (Khangarot, 1992; Chen et al., 2015; Xing et al., 2015), hypoxia (Beck et al., 2016), viral contamination (Liu et al., 2015), fasting, or nutritional imbalance (Seiliez et al., 2012; Yabu et al., 2012; Wei et al., 2017, 2018; Séité et al., 2018; Wang et al., 2018). In these last few years, increasing research also focused on the mechanisms involved in the control of this cellular function in fish, particularly in zebrafish (He et al., 2009; Dowling et al., 2010; He and Klionsky, 2010; Mathai et al., 2017). In contrast, surprisingly, its metabolic role remains poorly explored in these species and very little data is currently available on this subject.

However, we previously reported that rainbow trout treated with the autophagy flux inhibitor agent Colchicine exhibited severe alterations in hepatic carbohydrate and fat metabolisms, as revealed by a significant decrease in plasma glucose levels associated with a decrease of the concentration of some glucogenic amino acids in the liver, but also an increase in hepatic triglyceride and lipid droplet contents (Seiliez et al., 2016). Similarly, recent works provided the evidence for the degradation of lipid stores through lipophagy in the liver of fasted zebrafish (Wang et al., 2018) and yellow catfish (*pelteobagrus fulvidraco*) fed zinc supplemented diet (Wei et al., 2018). Together, these data are in close agreement with the aforementioned metabolic role of autophagy demonstrated in mammals (Madrigal-Matute and Cuervo, 2016). However, they also suggest that in addition to this previously reported role of autophagy in providing substrates for glucose production, energy furniture, or the synthesis of specific proteins, it could also play a major role in the regulation of the expression of some key metabolic genes. Indeed, these studies pointed out that autophagy inhibited fish exhibited strong perturbations in the mRNA levels of genes involved in hepatic carbohydrate and fat metabolisms (Seiliez et al., 2016; Wang et al., 2018). This would be an unknown function for autophagy. However, they show conflicting outcomes on specific gene expression regulations, precluding a clear picture of the role of autophagy in this process. Such differences could be explained by the divergence of experimental protocols such as the use of (1) different fish models (zebrafish vs. rainbow trout) and (2) different autophagy flux inhibitors (chloroquine vs. colchicine) and deserved further investigations.

In the present study, we treated primary cultures of trout hepatocytes with another autophagy flux inhibitor, the Baf A1, to assess the specificity of the previously reported *in vivo* effect of colchicine-mediated autophagy inhibition on the expression of several metabolism-related genes in this species. Baf A1 is widely used *in vitro* as an autophagic flux inhibitor. This drug inhibits the lysosomal V-ATPase to prevent its acidificationas well as the Ca2+ pump SERCA to disrupt autophagosome-lysosome fusion, together resulting in a strong block of autophagic flux (Mauvezin and Neufeld, 2015). The use of primary cultures of trout hepatocytes is an additional asset for our study, as they allow testing the response of the studied factors to specific stimuli independently of their systemic effects. This model is now widely used to improve understanding of intermediary metabolism in fish (Moon et al., 1985).

## Materials and Methods

### Animals

Sexually immature rainbow trout having a mean initial weight of 200 g were obtained from the INRA experimental facilities at Donzacq (Landes, France). Fish were maintained in tank kept in open circuits at a constant water temperature of 17°C, under natural photoperiod. They were fed to satiety every 2 days with a commercial diet (T-3P classic, Trouw, France). The experiments performed in the present study comply with the EUdirective 2010/63/EU on the protection of animals used for research as well as the decree No 2013-118, 1 February 2013 of the French legislation on the ethical treatment of animals.

### Hepatocyte Cell Culture

Rainbow trout liver cells were isolated from 3 days feed-deprived fish according to the previously detailed protocol (Lansard et al., 2010). We measured the cell viability (>98%) with trypan blue exclusion method (0.04% in 0.15 mol/L NaCl) and cells were counted using Neubauer chamber. They were then plated in a 6-well Primaria culture dish (BD) at a density of 3.106 cells/well and incubated at 18°C, the optimal temperature for cell cultures of trout origin, with complete medium containing modified Hanks’ medium (136.9 mmol/L NaCl, 5.4 mmol/L KCl, 0.8 mmol/L MgSO_4_, 0.44 mmol/L KH_2_PO_4_, 0.33 mmol/L Na_2_HPO_4_, 5 mmol/L NaHCO_3_, and 10 mmol/L HEPES) supplemented with 1% defatted BSA, 3 mmol/L glucose, 2% MEM essential amino acid mixture, 1% MEM non-essential amino acid mixture and 1% antibiotic antimycotic solution (1X) (sigma). The incubation medium was replaced every 24 h over the 48 h of primary cell culture. Microscopic examination ensured that hepatocytes progressively re-associated throughout culture to form cell heap. After 2 days of culture, the cells were incubated in a minimal medium deprived of serum and amino acids (a condition known to activate autophagy) in presence or absence of 100 nM of Baf A1 a concentration commonly used to block autophagosome-lysosome fusion *in vitro* (Klionsky et al., 2016). Cells were then sampled 4, 8, 16, and 24 h after the treatment and were prepared for western blot analysis or resuspended in TRIZOL reagent (Invitrogen, Carlsbad, CA, United States) and stored at -80°C for subsequent analyses. Each experiment was repeated 2 times.

### Protein Extraction and Western Blot Analyses

Cells were prepared for western blot analyses according to the previously detailed protocol (Lansard et al., 2010). LC3-II levels were measured by western blot as described previously in Belghit et al. (2014) and using the following antibodies: anti-LC3b (#2775 Cell Signaling Technology) and anti-TUBB (#2146, Cell Signaling Technology). These antibodies have already been validated in rainbow trout (Belghit et al., 2014).

### Quantitative RT-PCR Analyses

The protocol conditions for sample preparation and quantitative RT-PCR have been previously published (Lansard et al., 2010). The primers used for real time RT-PCR assays are listed in Table 1. Primer of *edem1* and *xbp1* were newly designed using Primer3 software. The primers that amplified glucose and lipid metabolism-related genes have already been described in previous studies (Plagnes-Juan et al., 2008; Marandel et al., 2015; Seiliez et al., 2016). For the expression analysis, relative quantification of target gene expression was done using the ΔCT method described by Pfaffl et al. (2002). The relative gene expression value of *eef1a1* was used for the normalization of the measured expression values of the target mRNA, and was found to not change significantly over sampling time or among treatments (data not shown).

**Table 1:** Sequences of the primer pairs used in the quantitative real-time RT-PCR assays.

Genes	Forward primer	Reverse primer
**Gluconeogenesis related genes**		
*pck1*	ACAGGGTGAGGCAGATGTAGG	CTAGTCTGTGGAGGTCTAAGGGC
*pck2*	ACAATGAGATGATGTGACTGCA	TGCTCCATCACCTACAACCT
*fbp1b1*	CTCTCAAGAACCTCTACAGCCT	TCAGTTCTCCCGTTCCCTTC
*g6pca*	GATGGCTTGACGTTCTCCT	AGATCCAGGAGAGTCCTCC
*g6pcb1*	AGGGACAGTTCGAAAATGGAG	CCAGAGAGGGAAGAAGATGAAGA
*g6pcb2*	CCTGCGGAACACCTTCTTTG	TCAATTTGTGGCGCTGATGAG
**Lipid metabolism related genes**		
*fas*	TGATCTGAAGGCCCGTGTCA	GGGTGACGTTGCCGTGGTAT
*plin2*	CATGGAGTCAGTTGAAGTCGTC	AATTTGTGGCTCCAGCTTGCC
*plin3*	GATGTCCAACACCGTCACAG	TCGATTTCCAACTCGTCCTC
**ER stress related genes**		
*chop*	CTGCACACGGTCTGGAGCTG	GGATCTCGTCTGGGATCAGGT
*edem1*	GAACATCCAAACGGGACAGT	TGAGAAGAGGGAGGGAGTCA
*xbp1*	CAACCCCGAGAACACAGTTT	AAGTGACACACGCTGTGGTC
**Reference gene**		
*eef1a1*	TCCTCTTGGTCGTTTCGCT	ACCCGAGGGACATCCTGTG


### Free Amino Acid Analyses

Free amino acid concentrations in hepatocytes were determined by ion exchange chromatography with a ninhydrin post-column reaction (L-8900 Amino Acid Analyzer, Hitachi High-Technologies Corporation, Tokyo, Japan).

### Statistical Analyses

Data are expressed as means ± SD. Normality was assessed using the Shaprio–test, while the equality of variances was determined using Levene’s test. When the normality and/or equal variances of data were respected, two-way ANOVA was used to detect significant differences. Following two-way ANOVA analysis, the Tukey test was used for *post hoc* analysis. For all statistical analyses, the level of significance was set at *P* < 0.05.

## Results

### Baf A1 Inhibits Autophagy in Trout Hepatocytes

**FIGURE 1:**
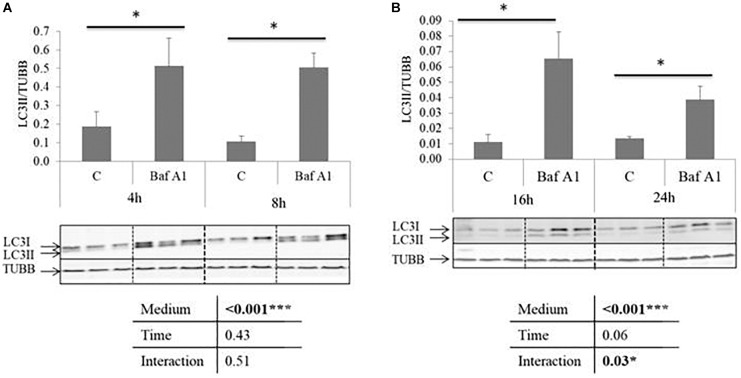
Baf A1 inhibits autophagy in trout hepatocytes. Representative LC3 and TUBB immunoblots of protein homogenates from trout hepatocyte treated with DMSO or Baf A1 for 4, 8. **(A)** 16 or 24 h **(B)**. Graphs show the ratio between LC3-II and TUBB used as a loading control. Values are means (*n* = 6), with standard error of the mean represented by vertical bars. ^∗^ was used to indicate significant difference between treatment (*P* < 0.05; two way anova test).

We first tested the ability of Baf A1 to block autophagy in our cell culture model. For this purpose, we analyzed by western blot the well-established autophagy marker LC3II in cells incubated in a serum- and amino acid-deprived medium (a condition known to activate autophagy) and treated or not with Baf A1 for 4, 8, 16, and 24 h. During autophagy, LC3 is converted from a non-lipidated cytosolic form (LC3-I) to a phosphatidylethanolamine-conjugated form (LC3II) on the autophagosomal membrane (Klionsky et al., 2016). However, LC3-II is also degraded during the late stage of autophagy, and it is now well accepted that the exposure of cells to lysosomal inhibitors, protease inhibitors or agent that block fusion of autophagosome with lysosomes, leads to LC3-II accumulation (Klionsky et al., 2016). As shown in Figure 1, the ratio of LC3-II to TUBB reached significantly higher levels in Baf A1 treated cells compared to non-treated cells. These results indicated that Baf A1 treated cells displayed a loss of autophagy function and that this drug is useful in our cell culture model.

### Baf A1 Treatment Affects the Expression of Key Genes of the Intermediary Metabolism in Trout Hepatocytes

**FIGURE 2:**
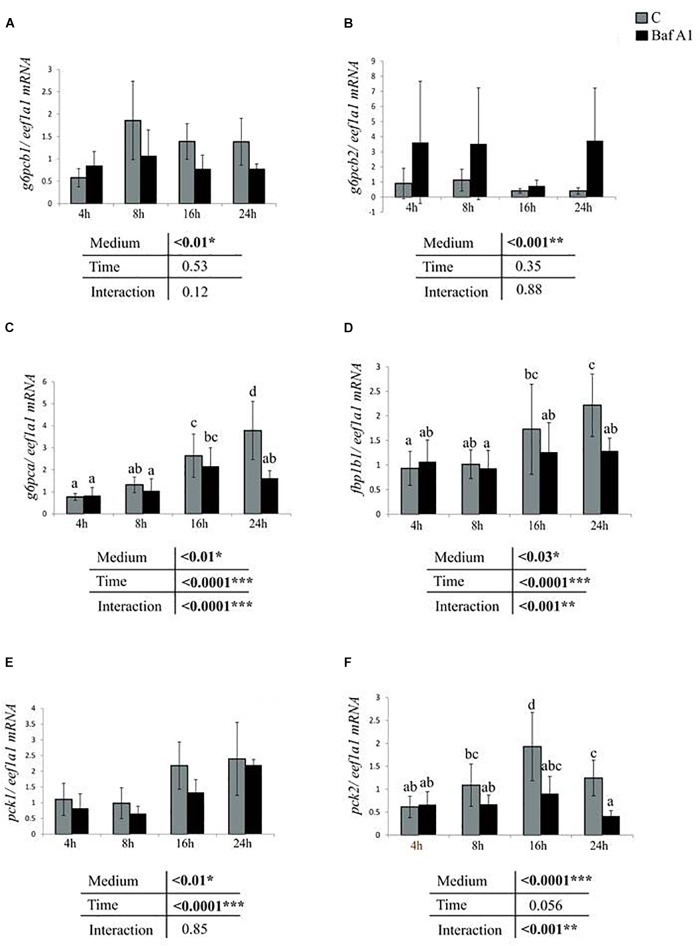
Baf A1 treatment affects mRNA levels of gluconeogenic genes. Hepatocytes were treated with DMSO or Baf A1 for 4, 8, 16, or 24 h. Hepatocyte mRNA levels of **(A)** *g6pcb1*, **(B)** *g6pcb2*, **(C)** *G6pca*, **(D)** *fbp1b*, **(E)** *pck1*, and **(F)** *pck2* were measured using quantitative real time RT-PCR assays. Expression values are normalized with the *eukaryotic translation elongation factor 1* ***α*** *1 (eef1a1)* mRNA. Value are means (*n* = 6) with standard error represented by vertical bars and were analyzed using two-way ANOVA (*P* < 0.05), followed by Tukey’s *post hoc* test for multiple comparisons. When interaction between sampling time and treatment is significant, lowercases letters (a, b, c, and d) represent statistically significant differences (*P* < 0.05, Tukey’s HSD).

We next addressed the consequences of Baf A1 treatment on the expression of several metabolism-related genes. We first monitored the expression of several genes of the gluconeogenesis in cells incubated in the same conditions described above with or without Baf A1. The obtained results showed that the addition of Baf A1 to the media led to a significant decrease of mRNA levels of gluconeogenesis-related genes *g6pcb1* and *pck1* regardless of the time of treatment (Figure 2A,E). Similar results were obtained for *g6pca* and *fbp1b1* at 24 h after the treatment (Figure 2C,D) and for *pck2* at 16 and 24 h after the treatment (Figure 2F). In contrast, we observed an increase of mRNA levels of one of the *g6pc* paralogs, the *g6pcb2*, in trout hepatocyte treated with Baf A1 (Figure 2B).

**FIGURE 3:**
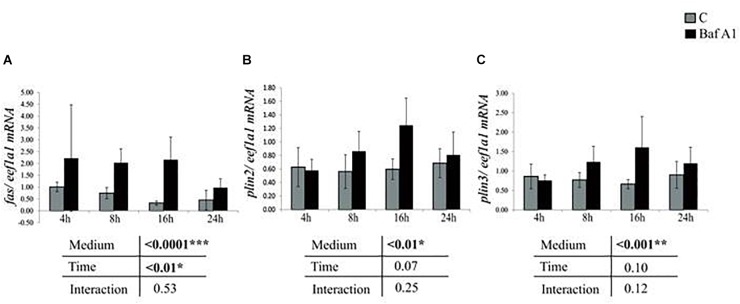
Baf A1 treatment induces mRNA levels of lipid metabolism related genes. Hepatocytes were treated with DMSO or Baf A1 for 4, 8, 16, or 24 h. Transcripts levels of **(A)** *fas*, **(B)** *plin2*, and **(C)** *plin3* were measured using quantitative real time RT-PCR assays. Expression values are normalized with the *eukaryotic translation elongation factor 1* ***α*** *1 (eef1a1)* mRNA. Value are means (*n* = 6), with standard error represented by vertical bars and were analyzed using two-way ANOVA (*P* < 0.05), followed by Tukey’s *post hoc* test for multiple comparisons.

We then analyzed the expression of several genes involved in lipid metabolism. The obtained results showed that mRNA levels of *fas* increased in cells treated with Baf A1 (Figure 3A). Similar results were obtained for *plin2 and plin3*, two critical regulators of hepatic neutral lipid storage (Figures 3B,C).

Overall, these data confirmed our previous *in vivo* results obtained with Colchicine and established a tight link between the activity of autophagy and the expression of several glucose and lipid metabolism-related genes.

### Baf A1 Treatment Lowers the Level of Free Amino Acids in Trout Hepatocytes

**FIGURE 4:**
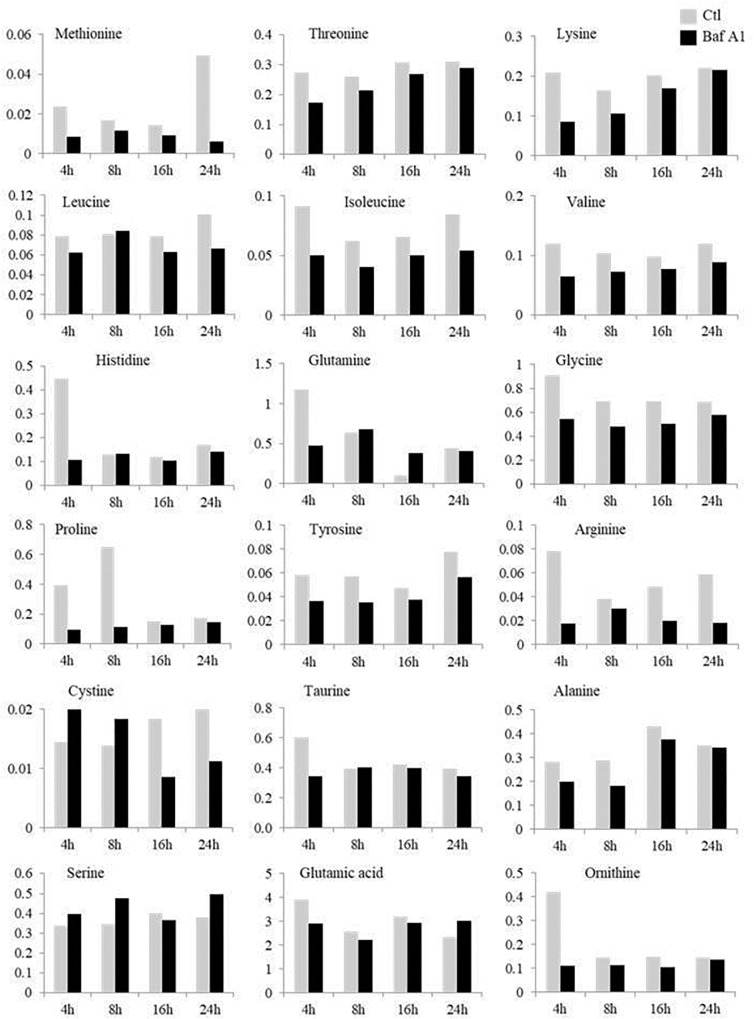
Time courses of the changes in free amino acids in fasted hepatocytes treated or not with Baf A1. Fasted trout hepatocytes were treated with DMSO or Baf A1 for 4, 8, 16, or 24 h. The concentration of each amino acid is expressed as μmol/l cell homogenate and was determined on a pool of samples from two independent experiments.

It is now well established that the expression of many metabolism-related genes is under the tight control of amino acid availability (Lansard et al., 2010, 2011). Autophagy being one of the main systems for the release of free amino acids during fasting, we wondered whether the effects of Baf A1 on metabolic gene expression could be related to a decrease in free amino acid levels in hepatocytes whose autophagy has been inhibited. We therefore, monitored the concentration of the main amino acids in fasted cells treated or not with Baf A1. As shown in Figure 4, hepatocytes treated with Baf A1 exhibited lower levels of most of the analyzed amino acids, in accordance with the reported role of liver autophagy on amino acid release during starvation. This global decrease in amino acid release in hepatocytes treated with BafA1 could therefore contribute to perturb the expression of the studied genes.

### Baf A1 Treatment Leads to ER Stress

**FIGURE 5:**
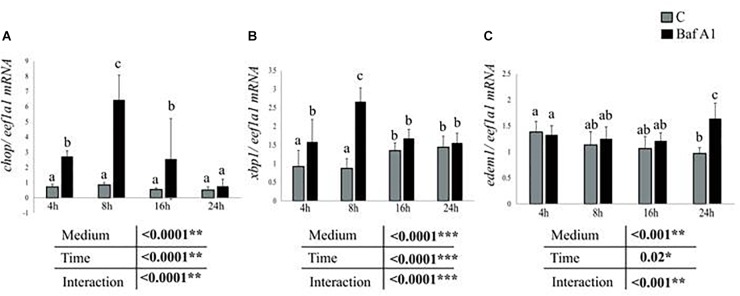
Baf A1 treatment induces ER stress markers. Hepatocytes were treated with DMSO or Baf A1 for 4, 8, 16, or 24 h. Hepatocyte mRNA levels of **(A)**, *chop* **(B)**, *xbp1*, and **(C)** *edem1* were measured using quantitative real time RT-PCR assays. Expression values are normalized with the *eukaryotic translation elongation factor 1 α 1 (eef1a1)* mRNA. Value are means (*n* = 6), with standard error with represented by vertical bars and were analyzed using two-way ANOVA (*P* < 0.05), followed by Tukey’s *post hoc* test for multiple comparisons. When interaction between sampling time and treatment is significant, lowercases letters (a, b, and c) represent statistically significant differences (*P* < 0.05, Tukey’s HSD).

Another hypothesis to explain the effect of Baf A1 on the expression of the studied genes concerns the endoplasmic reticulum (ER) stress. Accumulating evidences demonstrated that autophagy dysregulation causes ER stress (Yang et al., 2010), which has been shown to strongly impact the expression of intermediary metabolism-related genes (Lee et al., 2012; Wang and Kaufman, 2014; Zhou and Liu, 2014). However, to our knowledge, few if no data is available on the effect of autophagy dysregulation-mediated ER stress on the expression of intermediary metabolism-related genes. In the present study, we therefore sought to determine whether Baf A1 caused ER stress in our cells. To this end, we analyzed, in fasted hepatocytes treated with or without Baf A1, the expression of three target genes *chop, xbp1*, and *edem1* of the main ER-stress sensing pathways PERK, ATF6, and IRE1 pathways, respectively. As shown in Figure 5A, the mRNA levels of *chop* significantly increased in Baf A1 treated cells in comparison to control cells 4, 8 and 16 h after the treatment. Similar results were obtained for *xbp1*, with an increase at 4 and 8 h after the treatment (Figure 5B). Likewise, the mRNA levels of *edem1* increased 24 h after the treatment (Figure 5C), in line with previous findings demonstrating that *edem1* is a late ER-stress marker. Overall, the results obtained clearly show that hepatocytes treated with Baf A1 display sign of ER stress, which in turn could affect the expression of the studied genes.

## Discussion

Autophagy has long been considered merely as a cellular waste disposal and recycling mechanism. However, studies in recent years have highlighted its major role for the adaptation of metabolism to environmental changes (Kroemer et al., 2010; Chen et al., 2015; Chiarelli et al., 2016; Tang, 2016). In this regard, we and others showed that treatment of fasted fish (rainbow trout or zebrafish) with autophagy flux inhibitor agents (colchicine or chloroquine) led to strong defaults in intracellular substrates delivery for glucose production or energy furniture (Seiliez et al., 2016; Wang et al., 2018). Interestingly, these studies also pointed out sever perturbations in the mRNA levels of several intermediary metabolism-related genes in these fish, establishing a new potential link between autophagy and intermediary metabolism. However, probably due to divergences of experimental protocols (including the species investigated and the used autophagy flux inhibitors), these studies led to conflicting results with respect to the regulations of specific gene expression, precluding a clear picture of the role of autophagy in this process.

In the present study, we demonstrated that Baf A1 treatment of trout hepatocytes decreased the mRNA levels of genes involved in gluconeogenesis and conversely, increased those of genes involved in lipogenesis and lipid storage. Although it is well accepted that Baf A1 is an autophagy inhibitor, it may also have other side effects. For instance, some data reported that it has some effects on mitochondria quality (Yuan et al., 2015; Redmann et al., 2017), making it difficult to determine which effects on metabolism-related mRNAs could be a consequence of inhibiting autophagy or of direct effects on mitochondria independently of autophagy. However, our results are in close agreement with those previously reported in trout showing that *in vivo* treatment with colchicine (which act on autophagy by inducing microtubule disassembly) led to a similar lowering effect on the mRNA levels of gluconeogenesis-related genes and an increasing effect on both *plin2* and *plin3* (Seiliez et al., 2016), suggesting that the observed effects are specific to autophagy inhibition. Interestingly, previous findings in mammals also evidenced a tight link between the activity of autophagy and mRNA levels of some enzymes involved in glucose metabolism (Wang et al., 2015). Acute suppression of autophagy with lysosome inhibitors (Chloroquine or Bafilomycin A1) in statin treated human liver cancer cell line (HepG2 cells) has thus been shown to reduce mRNA levels of the two gluconeogenic enzymes *g6pc* and *pck1* (Wang et al., 2015). Similarly, the statin-induced increase in expression of *g6pc* and *pck1* was blocked in Atg7-deficient hepatocytes, providing a genetic confirmation of these results (Wang et al., 2015). However, another study suggests the opposite role of autophagy in gluconeogenesis with the finding that overexpression of Atg7 reduces mRNA levels of *g6pc* and *pck1* in the livers of mice (Yang et al., 2010); But the induction of autophagy by Atg7 overexpression was not verified in this study, preventing to conclude on the specific role of this function in the observed effects. More recently, Wang et al. (2018) showed that chloroquine treatment of fasted zebrafish inhibited the hepatic expression of most genes related to lipid metabolism and conversely upregulated those of carbohydrates metabolism, making possible the existence of species-dependent effects of autophagy inhibition. Overall, these data support a close link between autophagy and the mRNA levels of metabolic genes, although the exact nature of this relationship, which likely depends on many factors (including the species studied and/or the protocol used to monitor this link), remains to be clarified.

It is now clearly established that the expression of a wide range of hepatic genes involved in the intermediary metabolism is under the control of amino acid availability. In trout hepatocytes, free amino acid addition to an amino acid-deprived medium has thus been shown to up-regulate the mRNA levels of gluconeogenesis-related enzymes (Lansard et al., 2010, 2011). As autophagy is described as one of the main amino acid provider during fasting (Kuma et al., 2004; Ezaki et al., 2011), we therefore hypothesized that the observed effect of Baf A1 treatment on the mRNA levels of metabolic genes could be due to a default in free amino acid release in autophagy-inhibited hepatocytes. Accordingly, Baf A1 treated cells exhibited lower levels of most of the analyzed amino acids compared to control cells. Such an effect of autophagy in providing amino acids endogenously to sustain mechanistic target of rapamycin complex 1 (mTORC1) signaling when extracellular amino acids are limited has previously been reported in C2C12 murine myotubes (Yu and Long, 2014), and could therefore be also at play in the observed effect of Baf A1 on the studied genes. Interestingly, the expression of *g6pcb2* which, in contrast to the other analyzed gluconeogenesis-related genes increased in Baf A1 treated hepatocytes, was previously shown to exhibit an opposite regulation by feeding different levels of proteins and by amino acids levels in hepatocytes compared to other *g6pc* paralogs (Marandel et al., 2015; Lucie et al., 2016), tipping the scale in favor of a default of autophagy-dependent release of amino acids in the observed Baf A1 effect. However, not all studied genes are known to be under the control of amino acids *per se*. This is particularly the case for the gene *fas*, whose expression has already been shown to be not directly affected by the addition of amino acids to an amino acid-free medium in trout hepatocytes (Lansard et al., 2010, 2011). Instead, it is possible that the induction of ER stress observed in autophagy-inhibited hepatocytes plays an important role in the observed effect of Baf A1 treatment on these genes. Indeed, previous studies have shown that ER stress plays a critical role in regulation of lipid metabolism (Sriburi et al., 2004; Bobrovnikova-Marjon et al., 2008; Oyadomari et al., 2008; Rutkowski et al., 2008; Kammoun et al., 2009; Zhang et al., 2014). According to these studies, ER stress leads to activation of the evolutionarily conserved UPR signaling system in order to restore ER homeostasis (Shen et al., 2004). Accumulating evidence shows that activation of the UPR pathways can modulate lipid metabolism by controlling the transcriptional regulation of lipogenesis and triglyceride storage (Basseri and Austin, 2012; Han and Kaufman, 2016). For example, PERK and eIF2α phosphorylation are induced by antipsychotic drugs, resulting in increased lipid accumulation in hepatocytes through activation of sterol regulatory element–binding proteins SREBP-1c and SREBP-2, two transcription factors that regulate the expression of critical enzymes involved in lipogenic pathways including *fas* (Gosmain et al., 2005; Lauressergues et al., 2012). XBP1 also seems to be involved in the lipid metabolism through both direct and indirect activation of the transcription of key lipogenic genes in the liver, including *fas*, *plin2* as well as *CoA, desaturase 1* (*Scd1*), *Dgat2*, and *Acc2* (Lee et al., 2008, 2012). Together, these data support a possible role of ER stress in the observed effect of Baf A1 on the mRNA levels of enzymes involved in lipid metabolism. Noteworthy, UPR signaling has also been shown to affect the expression of genes involved in glucose metabolism and more particularly those of the gluconeogenesis pathway (Wagner and Moore, 2011).

Finally, recent findings in mammals reported a novel RNA degradation system called RNautophagy, during which direct import of RNA into lysosomes followed by degradation takes place (Fujiwara et al., 2013). During this process, the putative nucleic acid transporter SIDT2 predominantly localizes to lysosomes and mediates the translocation of RNA into lysosomes (Aizawa et al., 2016; Contu et al., 2017). Interestingly, the authors found that treatment of cells with lysosome inhibitors (chloroquine or Bafilomycin A1) hindered the SIDT2 overexpression-mediated increase in intracellular RNA degradation. These data make therefore the impairment of RNautophagy a possible mechanism of the observed inducing effect of Baf A1 in the level of some mRNAs. However, it remains to be established whether or not RNautophagy or a RNautophagy-like process exists in fish.

### Wrapping-Up

In the present study, we report that the treatment of fasted trout hepatocytes with Baf A1 strongly perturb the mRNA expression of several genes involved in glucose and lipid metabolisms. These results are in close agreement with those already reported with other autophagy inhibitors both in mammals and fish, and support a tight link between autophagy activity and the mRNA levels of metabolic genes. The underlying mechanisms are likely multiple and highlight the complexity of the crosstalk between ER, autophagy and metabolism.

Interestingly, the observed decrease in mRNA levels of gluconeogenic genes in cells treated with Baf A1 is also consistent with the reported role of autophagy in the maintenance of blood glucose during fasting by releasing amino acids for glucose production via gluconeogenesis (Ezaki et al., 2011). Similarly, we observed an increase in mRNA levels of FAS and the two LD-associated proteins PLIN2 and PLIN3 in Baf A1 treated cells in agreement with the well-established role of autophagy in the control of lipid stores during fasting (Singh et al., 2009; Wang et al., 2018; Wei et al., 2018). Autophagy could thus combine its role as a supplier of substrates for the production of glucose or energy furniture with the molecular regulation of several related metabolic enzymes.

In the future, important issues will be to confirm these observations by establishing fish cell lines whose autophagy is genetically invalidated, which is now possible with the CRISPR-Cas9 technology. Gaining knowledge in the relationships between ER, autophagy and metabolism is of paramount for a better understanding of the mechanisms involved in metabolic adaptation of fish to environmental stresses.

## Author Contributions

SS, SP, and IS designed the research. TP, NO, and EP-J conducted the analyses. All the data were obtained and analyzed by TP, NO, and SS under the supervision of SP and IS. The manuscript was written by SS and critically revised by IS and SP. All authors have read and approved the final manuscript. IS had primary responsibility for final content.

## Conflict of Interest Statement

The authors declare that the research was conducted in the absence of any commercial or financial relationships that could be construed as a potential conflict of interest.

## Abbreviations

Acc2: acetyl CoA carboxylase 2
ATF6: activating transcription factor 6
Baf A1: Bafilomycine A1
chop: C/EBP homologous protein
CoA: stearyl coenzyme A
Dgat2: diacylglycerol acetyltransferase 2
edem1: ER degradation enhancing alpha-mannosidase like protein 1
eef1a1: eukaryotic elongation factor 1 α 1
eIF2α: eukaryotic initiation factor 2α
fas: fatty acid synthase
fbp: fructose 1,6-bisphosphatase
g6pc: glucose 6-phosphatase
IRE1: inositol requiring 1
pck1: phosphoenol pyruvate carboxykinase 1 (cytosolic)
pck2: phosphoenol pyruvate carboxykinase 2 (mitochondrial)
PERK: PKR-like ER kinase
plin2: perilipin 2
plin3: perilipin 3
Scd1: stearyl desaturase 1
SIDT2: SID-1 transmembrane family member 2
SREBP: sterol regulatory element–binding proteins
TUBB: β-tubulin
UPR: unfolded protein response
xbp1: X-box binding protein 1
